# Ultrahigh resolution and color gamut with scattering-reducing transmissive pixels

**DOI:** 10.1038/s41467-019-12689-2

**Published:** 2019-10-21

**Authors:** June Sang Lee, Ji Yeon Park, Yong Hwan Kim, Seokwoo Jeon, Olivier Ouellette, Edward H. Sargent, Dong Ha Kim, Jerome K. Hyun

**Affiliations:** 1 Department of Chemistry and Nanoscience, Ewha Womans University, Seoul, 03760 Republic of Korea; 2 R&D Center, KOS, Inc., Hanam, Gyeonggi-do 12930 Republic of Korea; 3 Department of Materials Science and Engineering, KAIST Institute for the Nanocentury, Korea Advanced Institute of Science and Technology (KAIST), Daejeon, 34141 Republic of Korea; 4 Department of Electrical and Computer Engineering, University of Toronto, Toronto, ON M5S 3G4 Canada; 5 Division of Chemical Engineering and Materials Science, College of Engineering, Ewha Womans University, Seoul, 03760 Republic of Korea; 6 State Key Laboratory of Molecular Engineering of Polymers, Fudan University, 200433 Shanghai, China

**Keywords:** Nanophotonics and plasmonics, Nanocavities, Imaging and sensing, Other photonics

## Abstract

While plasmonic designs have dominated recent trends in structural color, schemes using localized surface plasmon resonances and surface plasmon polaritons that simultaneously achieve high color vibrancy at ultrahigh resolution have been elusive because of tradeoffs between size and performance. Herein we demonstrate vibrant and size-invariant transmissive type multicolor pixels composed of hybrid TiO_x_-Ag core-shell nanowires based on reduced scattering at their electric dipolar Mie resonances. This principle permits the hybrid nanoresonator to achieve the widest color gamut (~74% sRGB area coverage), linear color mixing, and the highest reported single color dots-per-inch (58,000~141,000) in transmission mode. Exploiting such features, we further show that an assembly of distinct nanoresonators can constitute a multicolor pixel for use in multispectral imaging, with a size that is ~10-folds below the Nyquist limit using a typical high NA objective lens.

## Abstract (web summary)

Tradeoffs between size and performance have limited plasmonic structural color vibrancy at high resolution. Here the authors present a nanophotonic resonant metal-coated nanowire capable of being used as a size invariant, vibrant multicolor pixel.

## Introduction

The ability to filter vibrant colors within submicron spatial scales is key to achieving advanced display^1,2^, optical storage^3,4^, security encryption^2,5,6^, multiplexing^7,8^, bio-imaging/sensors^9^, and multispectral/hyperspectral imaging^10–12^ exhibiting enhanced spatial and spectral resolution. The filter needs to support the display of spectrally pure individual colors, access to a wide-gamut color space that includes highly vibrant or saturated colors, size-invariant response, maintenance of similar intensities among different colors, use of intuitive color mixing algorithms, and ability to independently control saturation and hue without disturbing the other. Filtering colors from micron and submicron pixels has already been demonstrated in transmissive^3,13–16^ and reflective^17–22^ designs. Many of these filters have relied on the use of surface plasmon polaritons (SPPs), which can be excited diffractively in metallic gratings, or the use of localized surface plasmons (LSPs) generated with metallic substructures. These designs provide tunable colors via structural control; however, a compromise must be made between color vibrancy and spatial resolution^23^ which challenges the filters from simultaneously satisfying the conditions described above. Plasmonic gratings involving the coupling of SPPs offer highly saturated colors, but lack spatial resolution because a large number of grating elements is needed to facilitate momentum matching between light and SPPs^13^. Moreover, since the period dictates color selectivity, mixing colors within a confined space is difficult and unintuitive. LSP-based metal substructures including nanodisks above a metal film provide sub-diffraction-limited resolution but lack color vibrancy because plasmon damping at the metal interface attenuates the quality factor of the mode^17^. Although such structures can achieve ultrahigh resolutions exceeding 100,000 dots-per-inch (DPI), they operate in reflection mode^20,24^, with applications aimed primarily toward displays or prints. One can question the need for such ultrahigh resolution in these cases since it exceeds the resolving power of the human eye by several orders of magnitude^25^, while the color gamut is poorer than that of standard commercial pixels.

Other designs that achieve ultrahigh resolution in structural colors have been demonstrated with dielectric Mie resonators in the form of Si nanowires^26^, nanoblocks^27,28^, and Cr/Si hybrid nanoblocks^29^. The large index contrast between resonator and air helps to prevent mode overlap between adjacent elements to achieve high DPIs but at the expense of introducing multipolar resonances that compromise the spectral purity. Furthermore, Si absorbs in the visible range, resulting in nontrivial optical losses.

Photonic (Mie) resonances have previously been investigated in horizontally-resting bare nanowires using photons^30^ and fast electrons^31^. When a metallic shell of an appropriate thickness is introduced, these resonances are strongly enhanced, enabling enhanced nonlinear signals in ZnO nanowires^32^. Scattering is also reduced, leading to the creation of a single Ag-ZnO-Ag hybrid nanowire color filter^33^ and an invisible photodetector based on a Si nanowire^34^. In the quasi-static approximation, complete scattering cancellation occurs when the electric field of the incoming light encounters a two-component core-shell nanoparticle or nanowire whose inner and outer components exhibit out-of-phase overall electric dipole moments of identical strengths^35^. At an appropriate geometric ratio of inner and outer components, the two opposing overall electric dipole moments effectively cancel and the incident wave passes through the hybrid structure undisturbed. Such a phenomenon can also be found from an exact analytical treatment of Mie scattering from a core-shell infinite cylinder analogue, derived early on by M. Kerker^36^.

Herein, we demonstrate vibrant, transmissive color pixels with ultrahigh spatial resolution, employing an electric dipolar Mie mechanism that removes the tradeoff between color vibrancy and size. Our color pixel consists of an assembly of horizontally aligned 1D Ag-TiO_*x*_-Ag nanoresonators that use resonant scattering reduction at the fundamental (electric dipole) resonance to filter light polarized along the long axis of the optical element (i.e., s-polarized light). Color selectivity is determined not by the period of an array nor is it determined by the coupled fields subtending the interfaces of two metallic substructures. Instead, we form an enhanced photonic resonance within the oxide core assisted by the metallic shell, corresponding to a spectrally pure fundamental mode. Exploiting the oxide-metal core-shell nanowire geometry, we show, in this work, one of the highest sRGB space coverages of ~74% corresponding to a wide gamut that includes high color vibrancy, and highest lateral resolution of 141,000 DPI reported for transmissive color pixels at a fixed polarization. The realization of ultrahigh resolution and color saturation further allows the nanoresonators to achieve linear multicolor mixing at confined spatial scales. These features are well suited for snapshot multispectral imaging filters, which are subject to stringent size requirements several times higher than that of the underlying pixel array.

## Results

### Vibrant and transmissive color pixels based on reduced scattering

**Fig. 1:**
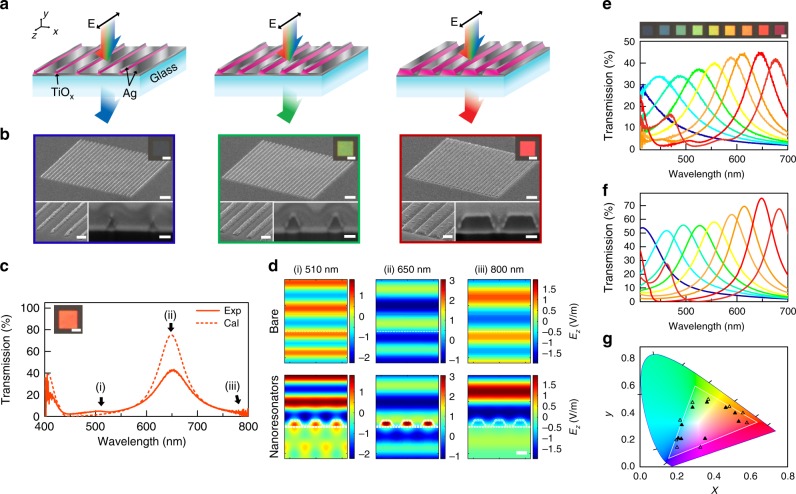
Color pixels from scattering-reducing nanoresonators. **a** Schematic of color pixels based on arrays of nanoresonators consisting of TiO_*x*_ nanowires enclosed by Ag layers. For normally incident s-polarized white light, each pixel transmits a distinct color determined by the nanoresonator width. The period is constant for all pixels. **b** From left to right, SEM images of blue, green, and red color pixels of increasing nanoresonator width but fixed period, acquired at a 63° tilt angle. Scale bar is 1 µm. Inset: OM images, SEM images of an enlarged view and FIB cross-sections of each pixel. Scale bar is 5 µm, 200 nm and 100 nm, respectively. **c** Measured and calculated transmission spectra of an orange pixel with nanoresonator width of 280 nm. Inset shows OM image and scale bar is 5 µm. **d** Calculated *z*-component of electric field distribution for bare glass substrate (top row) and for pixel from (**c**) (bottom row) at resonant (650 nm, middle column) and off-resonant (510 and 800 nm, left and right column, respectively) wavelengths. Scale bar is 200 nm. **e** OM images and measured transmission spectra of a pixel set, covering the full visible range. Scale bar is 5 µm. **f** Simulated transmission spectra of the pixel set. **g** CIE chromaticity diagrams of the pixel set. Solid and hollow triangles represent chromaticities derived from the measured and simulated transmission spectra, respectively. The white triangle depicts the sRGB color triangle. The deposited TiO_*x*_ film thickness is 115 nm

A schematic showing blue, green and red transmissive pixels (Fig. 1a) depicts an array of TiO_*x*_ nanowires sandwiched between two layers of Ag film, supported by a glass substrate (detailed fabrication process is described in the Methods section and Supplementary Fig. 1). The choice of the core dielectric arises from the need to minimize the nanowire size and optical loss for improved spatial resolution and color saturation, respectively. TiO_*x*_ exhibits a large real part and negligible imaginary part of the permittivity, satisfying both conditions. S-polarized light is normally incident from the top. In this example, the period between the nanowires was made constant for all pixels. The bottom and top Ag film thicknesses were 20 and 26 nm, respectively, which provided the optimum pixel contrast by balancing the quality of the photonic resonance of the nanoresonator with light leakage through the bare Ag background. The detailed role of each film is further described in Supplementary Figs. 2 and 3.

As shown schematically in Fig. 1a, the base width and height of the TiO_*x*_ nanowire determines the wavelength of the filtered light, whereby an increased width shifts the filtered color to a longer wavelength. SEM scans of the fabricated blue, green and red color pixels are shown in Fig. 1b from left to right respectively, imaged at a 63° tilt angle. Well-defined and regular nanoresonators are inside each pixel of 10 µm × 10 µm size. To evaluate the shape of the nanoresonators, we prepared cross-sections by FIB milling each pixel. SEM images of the cross-sections (bottom right inset of Fig. 1b) reveal trapezoidal shapes for wide nanowires (width above 310 nm) and triangular shapes for thin nanowires (width below 80 nm). We discuss this effect more in detail later, as it provides an important pathway for achieving full sRGB colors.

The filtering function of the fabricated pixels is depicted in Fig. 1c. As shown in the transmission spectrum of a representative, orange pixel in Fig. 1c, the color is dominated by a single peak in the visible range. The calculated and measured transmission spectra agree qualitatively, except near the deep-blue wavelength region because an increased uncertainty in spatial parameters of the calculated structure, modeled from cross-sectional SEM images, is introduced due to the decreasing size of the nanoresonator and finite resolution of the SEM. The measured spectrum also exhibits a lower peak intensity compared to calculation because of optical losses originating from a nontrivial Ag surface roughness.

The interaction between incoming light and nanoresonators that leads to reduced scattering and undisturbed propagation of light can be further understood by visualizing the *z*-component of the electric field distribution in the *x−y* cross-section of the pixel as shown in Fig. 1d. This distribution is compared to that of a bare glass substrate. For the pixel at nonresonant wavelengths (510 and 800 nm), the field is barely transmitted through the Ag film barrier, and is mostly reflected as can be observed from the intense field amplitude in air and weak amplitude in glass. This behavior contrasts from that of a bare glass substrate in which case reflection is trivial and transmission through the glass is efficient as can be seen by the negligible difference in field amplitudes between air and glass. At the resonant wavelength (650 nm), the nanoresonators behave as conduits for light, allowing power to squeeze through the Ag barrier (Supplementary Fig. 4) in a fashion similar to power flowing through subwavelength apertures in an opaque barrier. In fact, the localized transparency at each nanoresonator allows one to consider each nanoresonator as an effective aperture in the barrier (Supplementary Fig. 2d). When such apertures are spaced below the incident wavelength, all power transmits along the zeroth-order wave, rendering the barrier transparent. Indeed comparison of the field distribution between pixel and bare glass substrate at resonance reveals that the two respective fields behave similarly at all locations except at the nanoresonators. One can find intense fields concentrated at the center of each TiO_*x*_ nanowire, portraying signatures of an internal electric dipolar resonance rather than a plasmonic resonance. The strong spatial confinement of this fundamental mode underscores an important feature of the nanoresonators: the achievement of ultrahigh DPI without sacrificing the spectral characteristics of the resonance. The Ag shell not only enhances the resonance but also acts as an optical barrier to prevent mode overlapping among closely spaced adjacent nanoresonators (Supplementary Fig. 5), an effect that we will revisit.

As shown in Fig. 1e, the set of pixels under s-polarized white light illumination and viewed from the rear displays a full palette of visible colors. The transmission degrades severely under p-polarized illumination, as described in Supplementary Fig. 6. In this particular case, each pixel consists of identical hybrid nanoresonators arranged in an array with widths from 70 nm (blue) to 340 nm (red), interspaced at 150 nm. From the measured transmission spectra (Fig. 1e), each pixel presents a spectrally pure color, dominated by a single transmission peak, with the exception of the red pixel due to the emergence of a second-order photonic resonance. However, the second-order transmission peak is only 38% of the dominant fundamental peak in intensity, and therefore contributes weakly to the overall color production. For all pixels, the measured transmission efficiency stays within a range of 33−42%. The consistent intensities, and spectral purity qualitatively agree with the calculated spectra from all pixels (Fig. 1f). Chromaticities extracted from the measured and calculated spectra using the CIE 1931 chromaticity functions (see methods section) show that the colors span the full sRGB triangle (Fig. 1g).

Access to the full visible range was made possible by preserving the spatial symmetry of the fundamental mode across all nanoresonator widths. In other words, the TiO_*x*_ core varies in both lateral and vertical dimensions to accommodate the changing mode size. We employed a so-called resist closure effect in the TiO_*x*_ evaporation process to achieve this goal. Described in Supplementary Fig. 7, the effect refers to the gradual closing of the developed grooves during material evaporation due to the deposited material forming overhangs above the developed sites at low evaporation rates^37^. The resist closure effect is manifested in the structural shape of the deposited material. Exposure to material deposition from a shrinking opening results in the deposited structure assuming a shrinking width with increasing height. If the groove width is larger or smaller than the deposited thickness, structures with trapezoidal- or triangular-shaped cross-sections are produced, respectively. We experimentally demonstrated control over the range of resonances, or equivalently the color gamut, through the deposited TiO_*x*_ film thickness. At a deposited thicknesses of 68 nm, the pixel set displays a limited range of colors from blue to green (Supplementary Fig. 8). This range extends to include the red color when the deposited thickness increases beyond 92 nm. We plotted the chromaticities from the measured transmission spectra of three pixel sets with different deposited TiO_*x*_ film thicknesses to quantitatively evaluate the gamut range and associated color vibrancy (see Supplementary Fig. 8 and Supplementary Methods). Chromaticities positioned further away from the center of the sRGB triangle translate to higher color vibrancies. The measured chromaticities reside on the sRGB triangle borders, indicating a large gamut and full access to highly vibrant colors used in standardized display and imaging applications. We quantified the gamut by calculating the ratio of the accessible sRGB area to the full triangle area to be ~74%, corresponding to one of the largest color space coverages among transmissive nanoscale filters. If we consider a single pixel set made from a TiO_x_ film with deposited thickness of 92 nm, an sRGB gamut coverage of ~69% is achieved, still representing one of the largest values in transmission mode (Supplementary Fig. 9).

### Multicolor pixels via linear combinations of nanoresonators

**Fig. 2:**
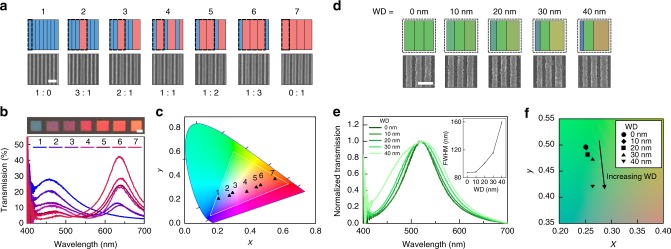
Multicolor pixels from linear combinations of nanoresonators. **a** Schematic (top) and SEM image (bottom) of a series of dual-color pixels, labeled from 1 to 7, composed of different ratios of two distinct nanoresonators. The dashed box represents the smallest repeating unit. Scale bar is 500 nm. **b** OM images and measured transmission spectra of dual-color pixels configured according to the series shown in (**a**) with the 1 and 7 pixel consisting of nanoresonators with widths of 100 and 250 nm, respectively. Scale bar is 5 µm. **c** CIE chromaticity diagram representing the chromaticities derived from the measured spectra of (**b**). **d** Schematic and SEM images of pixels for color saturation tuning. Each pixel consists of repeating units of three nanoresonators, where the outer two nanoresonators have width differences (WDs) of 0, 10, 20, 30, and 40 nm relative to the center nanoresonator. The width of the center element is fixed at 150 nm. Scale bar is 500 nm. **e** Normalized experimental transmission spectra from pixels with different WDs. Inset graph illustrates FWHM of transmission peak (i.e. color saturation) as a function of WD. **f** CIE chromaticity diagram of saturation-tuned pixels. Each marker represents the chromaticity attained with different WDs. The chromaticity shifts towards the center of CIE color space, indicating desaturated colors for increased WDs

The ability to predict and target arbitrary colors precisely through color mixing is critical in any imaging and display process. For transmissive colors involving RGB primaries, color mixing is additive; that is, distinct colors create a mixed color arising from the linear sum of each transmission spectrum. One of the challenges with SPP-based filters is the difficulty in meeting this rule, since plasmonic coupling between adjacent elements generates a nonlinear result. In our case, each nanoresonator tightly confines its photonic resonance in such a way that coupling is minimum at similar spacings. Therefore, assembling distinct nanoresonators in a confined space results in additive color mixing. Furthermore, the wide gamut, high color purity and consistent spectral forms offered by the nanoresonators facilitate the accurate production of any arbitrary color within the sRGB triangle. To demonstrate this concept, we designed 10 µm × 10 µm pixels consisting of “blue” and “red” nanoresonators with resonances at 450 and 640 nm, respectively, assembled at varying ratios as shown in Fig. 2a. Different ratios of nanoresonators used in each pixel labeled from 1 to 7 are depicted, where the dotted box represents the smallest repeating unit of distinct nanoresonators within the pixel. The SEM images show a section of representative pixels corresponding to the schematic. By controlling the ratio of the two distinct nanoresonators within the pixel, we modulate the spectral weight between the two respective transmission peaks relative to one another (Fig. 2b). As more red nanoresonators are added into the blue pixel at the expense of blue nanoresonators, the peak at 450 nm decreases in intensity while that at 640 nm increases, and vice versa. As shown in Fig. 2c, the measured chromaticities confirm that the nanoresonators indeed constitute a linear system (Supplementary Fig. 10), whereby combinations of the nanoresonators generate colors positioned along a straight line defined by the two pure colors of the two distinct nanoresonators. The same principle of additive color mixing can apply to any choice of two or more colors (Supplementary Figs. 11 and 12), enabling the creation of arbitrary and complex colors.

A key advantage of additive mixing with nanoresonators is the ability to sculpture the transmission response to a desired form. Figure 2d–f demonstrates an elementary example through the tunability of the full width half maximum (FWHM) of the transmission peak, corresponding to color saturation. A green pixel with a resonance at 510 nm was modified by replacing the outer two of three nanoresonators with slightly thinner and wider nanoresonators. Figure 2d describes schematically and with SEM images, the repeating units from five pixels fabricated by incorporating thinner and wider nanoresonators with width differences (WDs) of 0, 10, 20, 30, and 40 nm to the original nanoresonator (width = 160 nm). Because the negative and positive WD translates to a blue and red shift in peak position, respectively, that is much smaller than the FWHM of the transmission peak, the linear combination of the three nanoresonators results in the broadening of the original peak, as shown in the normalized transmission spectra of Fig. 2e. The inset of Fig. 2e plots the increase in FWHM of the transmission peak as a function of WD. One can see that the FWHM increases from 87.6 to 159.1 nm over a WD of 40 nm, corresponding to an increase of 182%. Consequently, the color desaturates as shown in the chromaticity diagram of Fig. 2f resulting in the loss of color vibrancy. Such results demonstrate a strategy for fine tuning the spectral shape of the filtered color.

### Size invariant color vibrancy and color mixing

**Fig. 3:**
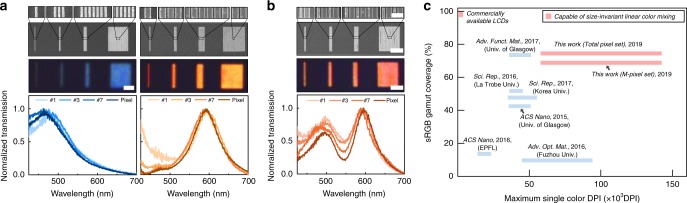
Ultrahigh resolution color pixels. **a** Characterization of single color pixels with varying size. Each pixel consists of 1, 3, 7, and more than 20 nanoresonators with widths of 80 nm (left column) and 250 nm (right column), interspaced at 100 nm. **b** Characterization of dual-color pixels with varying size. Each pixel consists of 1, 2, 3, and more than 20 repeating units of a pair of nanoresonators with widths of 100 and 250 nm, interspaced at 100 nm. **a**, **b** High-magnified SEM (first row), low-magnified SEM (second row), OM (third row) and normalized transmission spectra (fourth row) of the four pixels. Scale bar is 500 nm, 5 µm and 5 µm, respectively. **c** Plot of sRGB coverage and single color DPI from a representative collection of reported transmissive color filters. Color filters that are able to support size-invariant linear color mixing are shown in red. Note that all data points^3,8,14,16,50,51^ are calculated based on the information tabulated in Supplementary Table 1, except for those of commercially available LCDs^52^

By controlling the number of repeating units of nanoresonators, we vary the pixel size while preserving the high color vibrancy and additive color mixing schemes. Figure 3a illustrates the size-invariant performance of representative single-color pixels, where 1, 3, 7 and more than 20 identical nanoresonators comprise pixels with lateral dimensions of 80, 440, 1160 nm and ~10 μm, respectively for the blue pixels, and 250, 950, 2350 nm and ~10 μm, respectively for the orange pixels, as shown by the SEM images. The interspacing between adjacent nanoresonators was fixed at 100 nm. From the optical images and the normalized transmission spectra, one clearly sees that the hue and saturation are consistent among all four pixels for each color. Supplementary Figs. 13 and 14 show by calculation and experiment, respectively, similar size-invariant spectral characteristics for pixels of different colors. The size-invariant performance extends to mixed color pixels consisting of distinct nanoresonators. Figure 3b demonstrates an example using 100 and 250 nm-wide nanoresonators with resonances at 495 and 596 nm, respectively, interspaced at 100 nm. 1, 2, 3 and more than 20 repeating pairs of nanoresonators comprising pixels with lateral dimensions of 450, 1000, 1550 nm and ~10 μm generate similar responses regardless of pixel size, confirmed by the optical images and normalized transmission spectra. For both single- and dual-color pixels, a small resonance redshift can be found for the blue resonances when the pixel is reduced to a single nanoresonator. Although the Ag shell is known to prevent parasitic optical coupling between adjacent nanoresonators by tightly confining light within the core, close inspection of the resonant mode profile (Supplementary Fig. 5b) shows that the tails of the electric dipolar mode profile extend slightly outside the shell for the smallest (blue) nanoresonators. This is attributed to the relatively thinner Ag coatings on the sidewalls of the blue nanoresonators, as shown in the SEM cross-section images of Fig. 1b, leading to weaker confinement of the dipolar mode. Mode overlap between adjacent nanoresonators is therefore more pronounced for the blue resonances, leading to a small spectral shift. As shown in Supplementary Fig. 5b and Fig. 3a, b, however, Δ*λ*_res_/*λ*_res_ for both blue and red resonances between single element and array corresponds to a theoretical value of only 2.8 and 0.9% and measured value of 2.7 and 0.5% which translates to a negligible change in color production. This spectral shift can be minimized by thickening the Ag sidewall coating by a few nanometers, where calculations show that adding only ~3 nm to the Ag sidewall thickness reduces Δ*λ*_res/_*λ*_res_ to 1.1%, by strengthening the mode confinement.

Since the nanoresonator width and interspacing define the lateral size of the pixel, we calculate the single color DPI from the sum of the two parameters (Supplementary Fig. 14), resulting in a single-color DPI ranging from 58,000 (red) to 141,000 (blue). Supplementary Fig. 5 demonstrates that even at zero interspacing, representing the extreme case where the bases of neighboring nanoresonators make contact, the tight confinement of resonant light enables the spectral characteristics of the pixel to remain largely consistent, indicating that DPIs even higher than those found experimentally are theoretically achievable. Since most transmissive color filters are grating-type based on SPPs, the smallest pixel size generally requires at least two metallic elements between which a plasmonic gap forms. In this respect, the nanoresonator, which functions with a single element, provides two folds higher resolution. We compare the nanoresonators to other transmissive color pixels in Supplementary Table 1 by evaluating performance indicators such as the sRGB coverage ratio, absolute transmission efficiency for an array, periodicity independence, smallest pixel size, maximum DPI and size-invariant color mixing capability. Herein, the nanoresonator pixels achieve similar levels of intensity to other filters, while supporting additive color mixing capabilities. With an sRGB coverage ratio of ~74% for the total pixel set or ~69% for a single pixel set of the same deposited TiO_*x*_ thickness, and a resolution range of 58,000~141,000 DPIs, the nanoresonator pixels support one of the highest color vibrancies and resolutions among transmissive color filters operating at a fixed polarization, as summarized in Fig. 3c.

### Snapshot multispectral imaging filters

The above observations raise interesting possibilities for the use of nanoresonators as snapshot multispectral imaging filters. Multispectral imaging (MSI) refers to the acquisition of an image as a function of 2D position and wavelength, such that the spectral response, binned by several distinct spectral peaks, can be analyzed at every point of the image^38,39^. Two predominant MSI techniques exist^40^: the scanning type where 2D information is acquired while the wavelength is scanned via a variable color filter or other dispersive methods, and the snapshot type where 2D spatial and spectral information are acquired simultaneously through an array of color filters integrated with the detector array. So far, challenges with miniaturizing color filters down to sizes of the sensor elements (sensels) in the detector array has limited the snapshot type from achieving significant advances in resolution. Unlike typical color pixels that only use red, green and blue elements, MSI requires several filter elements to be incorporated into one pixel, restricting the filter size to a Nyquist limit that is several times smaller than that of the pixel array.

**Fig. 4:**
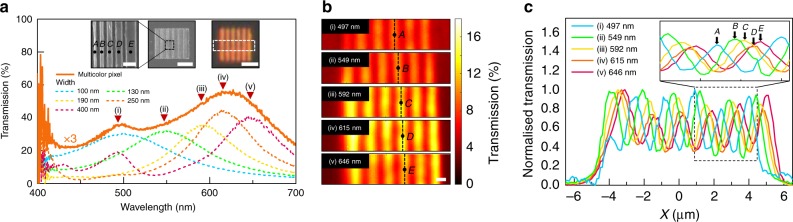
Snapshot multispectral imaging filters. **a** Transmission spectra of multicolor pixel (orange solid line, enhanced by threefolds) and the five constituent nanoresonators with widths (resonance) of 100 (497), 130 (549), 190 (592), 250 (615), and 400 nm (646 nm) (colored dotted lines) interspaced at 1500 nm. Resonances are marked by inverted red triangles. Inset: high- and low-magnified SEM images and OM image of multicolor pixel. From narrowest to widest width, the nanoresonators are labeled sequentially as *A*, *B*, *C*, *D*, and *E*. Scale bar is 500 nm, 5 µm and 5 µm, respectively. **b** 2D transmission maps at the five resonant wavelengths of (**a**), demonstrating the resolvability of the respective nanoresonators. Scale bar is 1 µm. **c** 1D normalized transmission intensity profile along the *x*-position at the five resonant wavelengths of (**a**). Enlarged intensity profile of representative segment confirms the sequence of positions of the *A*, *B*, *C*, *D*, and *E* nanoresonators

Displaying one of the highest color vibrancies and spatial resolutions, our nanoresonators offer a promising platform for meeting this strict spatial band limit. Figure 4 elucidates this ability in more detail through the spatial and spectral characterization of a multicolor pixel. We fabricated a multicolor pixel consisting of five distinct nanoresonators with widths (resonances) of 100 (497), 130 (549), 190 (592), 250 (615), and 400 nm (649 nm), respectively. When illuminated by a plane wave, each nanoresonator features a distinct transmission peak that combines to produce a broad transmission spectrum, as shown in Fig. 4a. In practice, incident light would be focused onto the MSI pixel through a low NA lens. Supplementary Fig. 15 shows that the transmission spectrum does not change significantly in such case. SEM images of the multicolor pixel show the sequence of nanoresonators, where *A*, *B*, *C*, *D*, and *E* denote each element (Fig. 4a inset) in the order of thin to wide nanoresonators, respectively. The corresponding OM image of the pixel shows intensity modulations across the lateral dimension of the pixel (Fig. 4a inset). These features are understood more clearly through spatial transmission maps acquired at each resonant wavelength using a 0.9 NA objective lens. As shown in Fig. 4b, each nanoresonator is clearly resolvable at its respective resonant wavelength despite the fact that the spacing between adjacent nanoresonators is below the diffraction limit.

To observe the absolute positions of the nanoresonators, normalized line profiles corresponding to each resonance, across the lateral dimensions, are presented in Fig. 4c. The measured width of each nanoresonator is a convolution of the resolution of our imaging system and the actual nanoresonator width. The number of peaks at every resonant wavelength matches exactly the number of repeating units in the optical and SEM images. Furthermore, the spatial sequence of distinct nanoresonators correspond to the designed sequence, where wider nanoresonators are located at larger *x* positions. The shifting position markers denoting the *A*, *B*, *C*, *D*, and *E* nanoresonators in Fig. 4c also confirm the sequence. The measured center-to-center spacing between adjacent nanoresonators of 200~250 nm roughly corresponds to that obtained by SEM, with the exception of the spacing between the *A* and *B* nanoresonators (~500 nm). This is attributed to the fact that the signal from the *A* nanoresonator contains components of the *E* nanoresonator as the resonance overlaps with the second-order resonance of the *E* nanoresonator.

The above results show that each nanoresonator filters color reliably at its respective wavelength and position. We observe spatial and spectral filtering in the range of 497~646 nm over a length of 1.67 μm. Using a typical high-resolution 100 × 0.9 NA objective lens, this corresponds to a pixel size that is one-order of magnitude below the Nyquist limit (16.8~21.9 μm). Such properties open up intriguing possibilities for achieving ultrahigh-resolution snapshot multispectral imaging by integrating the filter to a spectrally resolved detector array (SRDA)^7,41^ that could be formed with the hybrid nanoresonators themselves using individually addressable Ag contact designs and doping schemes. We note that the spatial resolution in the lateral dimension may also be improved by decreasing the nanoresonator aspect ratio, but at the expense of intensity and color saturation (Supplementary Fig. 16).

It is worth noting that our MSI design exhibits spectrally overlapping bands, which in conventional MSI/hyperspectral image (HSI) processing may limit the spectrum reconstruction ability. One solution is to use a postprocessing algorithm that processes information from the spectral band overlap to form an adapted band of narrower width than that of the original band of each nanoresonator^42^. Originally developed by researchers of quantum-dot infrared photodetectors^43–45^, such an algorithm can be applied to a linear system of photodetectors such as the SRDA-nanoresonator filters that we envision. It can be shown that a desired narrowband response can be approximated by a weighted superposition of the original spectral bands, resulting in an adapted response. The weighting coefficients used therein can then be applied to the measured photocurrents from each nanoresonator photodetector to yield the photocurrent that would be obtained from the adapted spectral response^42^. To achieve even narrower adapted responses and a broader accessible spectral range, the algorithm requires more distinct nanoresonators (i.e. more bands). However, this inevitably increases the minimum pixel size, decreasing the spatial resolution. Therefore, further improvements in the MSI spectral resolution and range must be met with a concomitant decrease in spatial resolution. To reduce this tradeoff, we can replace the dielectric material with other higher index materials, optimize the resist-closure effect (see Supplementary Fig. 7) to obtain a larger range of resonances with smaller nanoresonator widths, or strengthen the mode confinement by increasing the Ag sidewalls thickness (see Supplementary Fig. 3) which also helps minimize the small size-variance in resonant wavelengths for the deep-blue bands as discussed previously.

## Discussion

Herein, we have demonstrated transmissive color pixels that support vibrant colors at ultrahigh resolutions using resonantly reduced scattering enabled by metallic shell-enhanced electric dipolar resonances. The unique operational principle permits the nanoresonator to perform as a dedicated filter, with the hue, saturation and size independently controllable. The decoupled parameters enable the pixel to achieve one of the largest sRGB coverage ratios of ~74%, precise additive color mixing capabilities, and highest resolution range of 58,000 to 141,000 DPI among transmissive color filters. We further exploited the superior performance indicators to explore their potential as snapshot MSI filters. Five distinct nanoresonators were spatially and spectrally resolved at their respective positions spanning 1.67 μm, and resonances ranging from 497 to 646 nm. Such a size is ~10-folds below the Nyquist limit defined by a 100 × 0.9NA objective lens, demonstrating potential for ultrahigh-resolution snapshot MSI technologies. These results provide an alternative pathway to plasmon-based systems for exploring opportunities and addressing challenges in the field of structural colors.

## Methods

### Device fabrication

Ge and Ag of sub-1 nm and 20 nm thicknesses were sequentially deposited onto a clean glass substrate with an e-beam evaporator (EBX-2000, ULVAC), with deposition rates of 0.02 and 0.04 nm/s, respectively. Standard e-beam lithography was performed on a 300-nm-thick PMMA on the substrate, with 1 nA beam current (beam size: 5 nm), 100 keV acceleration voltage and 700 µC/cm^2^ dose. After development, TiO_*x*_ of thicknesses ranging from 70 to 120 nm were deposited onto the patterned substrates via e-beam evaporation, with deposition rates of 0.06 nm/s. Three sets of pixels were fabricated with TiO_*x*_ thicknesses of 68, 92, and 115 nm, denoted as L (low-), M (middle-) and H (high thickness) pixel sets, respectively, to provide nanoresonator width-height relations for variable amounts of deposited TiO_*x*_. The substrate was subjected to a lift-off process followed by sequential deposition of Ge and Ag layers of sub-1 nm and 26 nm thickness, respectively, via e-beam evaporation. Ge layer of sub-1 nm thickness serves as a wetting layer to minimize the surface roughness of the deposited Ag layer^46^. The actual dimensions of the nanoresonators measured from the SEM images were found to have an error of ±10% compared to the designed dimensions. Experimental results of the H pixel set are shown in Fig. 1e–g, and those of the L and M pixel sets are described in Supplementary Fig. 8.

### Optical characterization and 2D transmission mapping

Transmission spectra of the pixels were measured using a 0.55 NA and 0.9 NA objective lens on a confocal microscope coupled to a spectrometer (SP-2300i, Princeton Instrument) through an optical fiber (M43L02, Thorlabs) of 105 µm diameter. Spectra were acquired from the center of the pixel. The size of the pinhole projected over the sample plane was about 2 µm which is smaller than the pixel size, thereby excluding contributions from the bare Ag background. The sample was illuminated by a white light source (MWWHL3, Thorlabs) with a linear polarizer (54-926, Edmund optics). The transmission intensity was normalized by the transmission spectrum of a bare glass substrate measured under the same setup. A digital CCD camera (Infinity lite, Lumenera) was employed to capture OM images with a 0.45 NA objective lens.

For the 2D transmission maps, the transmission spectrum was collected at every scan point using a scanning-stage (HE01BX, Prior Scientific Instruments) with step sizes of 100 nm in *x* and *y* directions, and using a 0.9 NA objective lens. An integration window of 4 nm was employed to map the transmission intensity at wavelengths of interest.

### FDTD modeling

Numerical simulations were carried out with a commercial FDTD software package (Lumerical FDTD, Lumerical Solutions) to calculate the zeroth-order far field transmission efficiency, electrical field, and power distribution. A 2D model obtained by fitting an analytical equation to the geometrical parameters measured from FIB cross-section images was employed for the calculations. A plane wave source was mainly employed in the simulation, except for Supplementary Fig. 15 that was performed with a one-dimensional (1D) Gaussian beam source. A 3D model was also employed for Supplementary Fig. 16 with a plane wave source and a 2D Gaussian beam source. The dielectric function for Ag was obtained from Johnson and Christy^47^, Ge from Palik^48^. That for TiO_*x*_ was measured from ellipsometry on a film prepared through e-beam evaporation. Slight discrepancies between experimental and simulated transmission spectra are associated with uncertainties in the thickness of Ge layers and the presence of Ag surface roughness.

### CIE color-matching function

The chromaticities of different pixel colors were calculated using the standard color-matching functions defined by the International Commission on Illumination (CIE)^49^. The CIE tri-stimulus values (*X*, *Y*, *Z*) were computed by integrating *T*(*λ*) with the CIE1931 2° standard observer functions $\left( {\bar x\left( \lambda \right),\bar y\left( \lambda \right),\bar z\left( \lambda \right)} \right)$ over the visible range through the following equations, where *T*(*λ*) is the measured/simulated transmission spectra.

1 $$X = {\smallint} {T\left( \lambda \right)\bar x\left( \lambda \right){\mathrm{d}}\lambda },$$

2 $$Y = {\smallint} T \left( \lambda \right)\bar y\left( \lambda \right){\mathrm{d}}\lambda,$$

3 $$Z = {\smallint} {T\left( \lambda \right)\bar z\left( \lambda \right){\mathrm{d}}\lambda },$$

The chromaticity coordinates (*x*, *y*, *z*) were computed from the tri-stimulus values as:

4 $$x = X/(X + Y + Z),$$

5 $$y = Y/(X + Y + Z),$$

6 $$z = 1 - x - y.$$

The calculated chromaticity coordinates of different pixels were illustrated on the (*x*, *y*)-plane of the CIE 1931 color space.

## Supplementary information

Supplementary Information

## Data Availability

The data that support the findings of this study are available from the corresponding authors upon reasonable request.
