# Supplementary material for: Ultrahigh resolution and color gamut with scattering-reducing transmissive pixels
Source: Nat Commun. 2019 Oct 21;10:4782. doi: 10.1038/s41467-019-12689-2 (PMC6803669; doi:10.1038/s41467-019-12689-2)
Supplement: Supplementary file 1 — Supplementary Information [file 41467_2019_12689_MOESM1_ESM.pdf]

Supplementary Information

for

“Ultrahigh Resolution and Color Gamut with Scattering-Reducing Transmissive Pixels”

Lee et al.

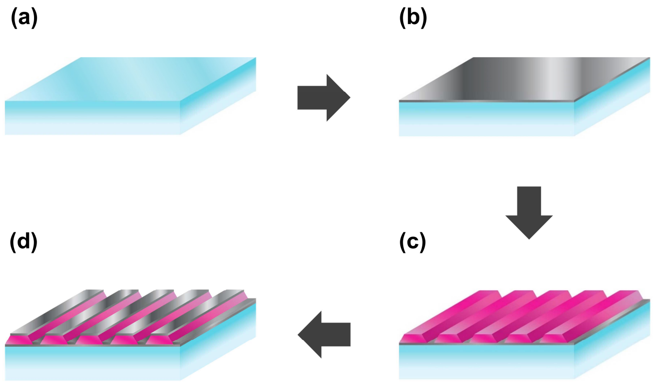

Supplementary Fig. 1. Fabrication process of color pixels. (**a, b**) Ge and Ag of sub-1nm and 20 nm thickness are sequentially deposited onto bare glass substrate via e-beam evaporation. (**c**) Standard e-beam lithography, development, liftoff and evaporation processes are performed to create arrays of TiO_x nanowires of different widths and heights. (**d**) Ge and Ag of sub-1nm and 26 nm thickness are sequentially deposited onto TiO_x nanowires.

Supplementary Note 1. Role of Ag films in resonant transmission through hybrid nanoresonators

To illustrate the role of the top Ag layer, we present the calculated scattering efficiency and full-wave simulations for three simple scenarios: a bare opaque Ag substrate, a nanoresonator without the top Ag coating on the Ag substrate, and a hybrid nanoresonator on the Ag substrate. Detailed structures are illustrated in the index profiles in Supplementary Fig. 2b. As displayed in Supplementary Fig. 2a, the bare Ag substrate does not scatter whereas the bare nanoresonator scatters over the wavelength range of interest. However, the hybrid nanoresonator with a 30nm-thick top Ag layer produces a sharp decrease in the scattering intensity at a resonant wavelength (633 nm). To further visualize the dramatically reduced backscattering of the hybrid nanoresonator, the z-component of the electric field under planar illumination is calculated at off-resonant (550 nm) and resonant (633 nm) wavelengths and compared with that of the bare Ag substrate and bare nanoresonator. One can see that the bare Ag substrate reflects all incident waves without any scattering, and that the bare nanoresonator scatters waves, observable through wave interference patterns in the air. The hybrid nanoresonator, on the other hand, shows minimal backscattering at the resonant wavelength (633 nm), in contrast to the scattering behavior of the off-resonant wavelength (550 nm). Here, the Ag coating introduces a dipole moment opposite to that of the internal dipole moment in the TiO_x, but equal in strength. Therefore the net dipole moment is nulled, cancelling the scattering. We can visualize the strong internal dipolar response as seen in the field distribution.

The bottom Ag film plays the simple role of effectively converting the reduced backscattering from an opaque Ag substrate into narrowband transmission through the hybrid nanoresonator. To demonstrate this, we present three scenarios with a thin Ag film on glass: a bare Ag thin

film, an open aperture in the thin film, and a hybrid nanoresonator on the thin film.

The Ag film thickness needs to be thin enough so that the hybrid nanoresonator can locally transmit light at resonance, but also sufficiently thick enough to ensure an optically opaque background for enhanced pixel contrast. We find that at an optimized thickness of 20 nm, transmission is sufficiently reduced for a bare Ag layer, while the hybrid nanoresonator resonantly transmits at a wavelength similar to the one observed for reduced backscattering described above (Supplementary Fig. 2c). Full wave simulations give some more insight into this picture. Because of dramatically reduced scattering, the hybrid nanoresonator effectively appears invisible to the incoming wave, and therefore behaves as a transparent aperture that gives rise to propagating wavelets in the transmitted region. In fact, this is analogous to the scenario where a plane wave hits an aperture of identical size to the hybrid nanoresonator, in the Ag film, as can be seen in Supplementary Fig. 2b. The bottom Ag layer therefore ensures light at resonance to transmit through by contributing to the formation of a dipolar resonance that makes the nanoresonator transparent.

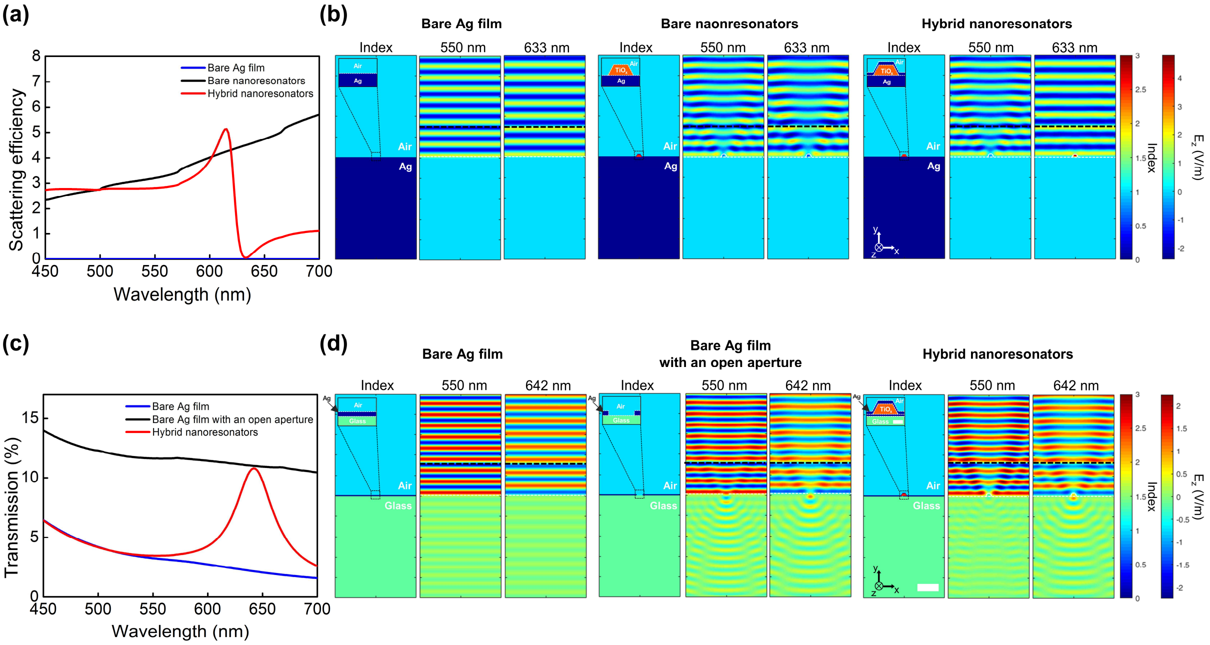

Supplementary Fig. 2. Scattering and transmission response of hybrid nanoresonator on Ag substrate and Ag thin film. (**a)** Calculated scattering efficiency as a function of wavelength for scenarios involving a bare Ag substrate. (**b**) Real part of the calculated E_z distribution at off-resonant (550 nm) and resonant wavelengths (633 nm) for a bare Ag substrate, bare nanoresonator on Ag substrate, and hybrid nanoresonator on Ag substrate. (**c**) Calculated transmission as a function of wavelength for scenarios involving Ag thin film on glass. (**d**) Real part of the calculated E_z distribution at off-resonant (550 nm) and resonant wavelengths (642 nm) for bare Ag thin film, open aperture in bare Ag thin film, and hybrid nanoresonator on bare Ag film. Top and bottom Ag layers are 30 and 20 nm thick, respectively. Scale bar is 1 μm. Inset: Enlarged index profiles and scale bar is 100 nm.

We extend the analysis of the single nanoresonator to that of an assembly of nanoresonators to further verify their optical responses as a function of Ag layer thickness. Since the top Ag layer plays a dominant role in defining the cavity, we vary the top Ag layer thickness**.** We calculated the zeroth order transmission and total reflection for the nanoresonator assembly with a period of 400 nm for various top Ag layer thicknesses (0 nm - 40 nm), as demonstrated in Supplementary Fig. 3. As the top Ag layer thickness increases, the resonant behavior of both transmission and reflection becomes more pronounced and blue-shifted, where transmission peaks are always in excellent agreement with reflection dips to keep the total power constant. The enhanced transmission for increased thickness also suggests improved cancellation of the internal (TiO_x) and outer (Ag) dipole moments in each hybrid nanoresonator. However, as the thickness is increased over 30 nm, the Ag shell becomes more optically opaque causing the absolute zeroth order transmission to drop.

We additionally visualized E_z to clearly depict the resonantly enhanced transmission and reduced backscattering for the hybrid nanoresonator array with varying Ag shell thickness. With the exception of the bare nanoresonators, all hybrid nanoresonators give rise to strong contrast in field amplitudes between glass and air at resonant wavelengths (middle column). The transmission is optimized for a 30 nm-thick top Ag shell.

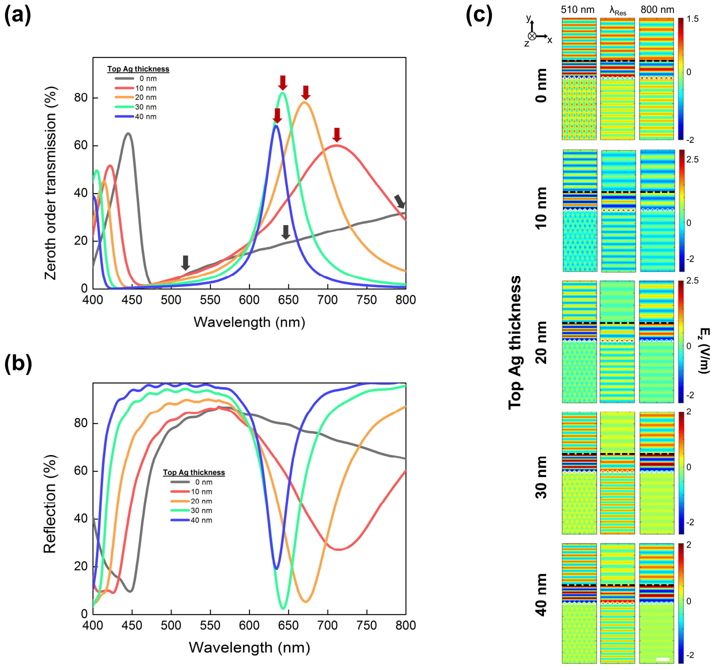

Supplementary Fig. 3. Optical properties of assembly of hybrid nanoresonators for varying top Ag layer thickness. (**a**) Zeroth order transmission and (**b**) total reflection of 280 nm-sized hybrid nanoresonators as a function of wavelength and top Ag layer thickness from 0 nm to 40 nm. (**c**) Real part of E_z at resonant and off-resonant (510 and 800 nm) wavelengths for different top Ag layer thicknesses. Scale bar is 1 µm

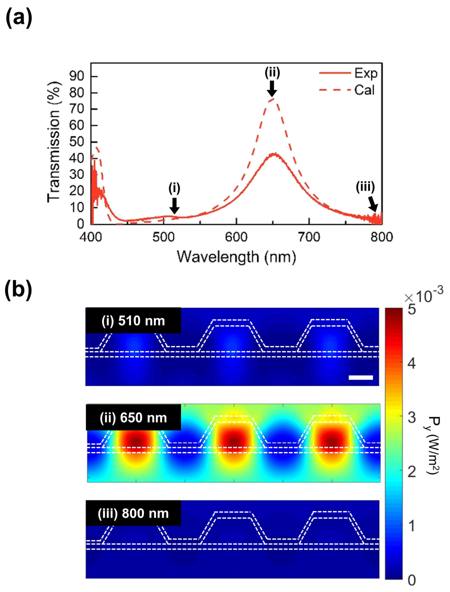

Supplementary Fig. 4. Power transfer through nanoresonators with width of 280 nm and periodicity of 400 nm (**a**) Measured and calculated transmission spectra of pixel. (**b**) Calculated distribution of real part of y-component (along optical axis) of Poynting vector for pixel at resonant (650 nm) and off-resonant (510, 800 nm) wavelengths. Scale bar is 100 nm.

Supplementary Note 2. Period dependent optical characteristics of nanoresonators

We consider excessively large periods (i.e., 1000 nm) to explore the response of single elements. Although the transmission decreases for larger periods as expected since the nanoresonator coverage decreases (Supplementary Fig. 5b), we emphasize an important aspect of this study in that the spectral position of the transmission peak stays fixed regardless of period and that the absolute transmission can be passively controlled (Supplementary Fig. 5c and d).

We also highlight the strong spatial confinement of the fundamental cavity mode as another important and useful feature of the hybrid nanoresonator for achieving ultrahigh DPIs. Supplementary Fig. 5b shows the tightly confined spatial profile of the fundamental mode in all three hybrid nanoresonators. As described above in the previous comment, the Ag coating assists in tightly confining light at resonance. One can see that the spectral peak shift is not significant up to the period where the two resonators make contact (dashed lines in Supplementary Fig. 5b and c). Larger peak deviations can be observed for the blue pixels due to increased coupling between weakly confined dipolar modes, caused by the thinner Ag coatings on the sidewalls. Once the period fall below the lateral size of the nanoresonator (i.e., adjacent resonators merge), enhanced coupling can be observed that dramatically redshifts the spectral peak. At the limit where the nanoresonators have merged into a three-layer film (Ag-TiO_x-Ag), the spectral peak converges to that of a planar Fabry-Perot resonator.

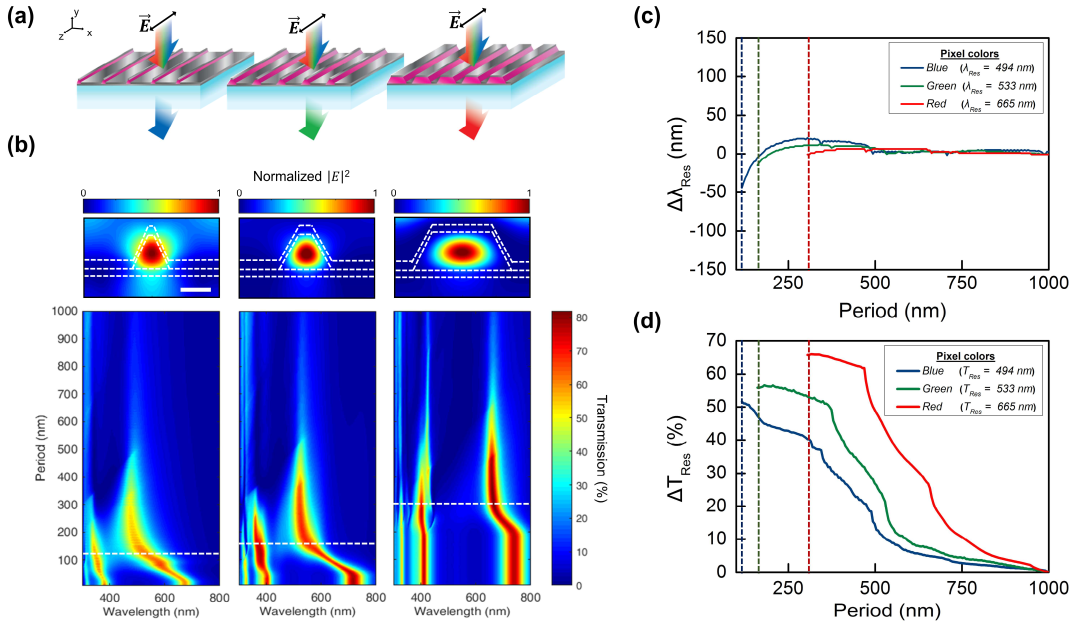

Supplementary Fig. 5. Transmission response as a function of period. (a) Schematic of blue, green and red pixels. (b) Calculated electric field distribution from unit cell and calculated transmission as a function of wavelength and grating periods, shown from left to right for blue, green and red pixels, respectively. Scale bar is 100 nm. (c) Resonant wavelength difference ($\Delta\lambda_{Res}$) and (d) Maximum peak transmission difference (ΔT_{Res}) between array and single nanoresonator for blue, green and red pixels as a function of period. Dashed lines depict the regime where interspacing between nanoresonators is 0 nm.

Supplementary Note 3. Non-plasmonic color filtering mechanism

A few key distinctions from grating-type SPP-based filters can be identified. First, the filtering mechanism is enabled when the electric field of the incoming light is s-polarized, as shown in Figure 1a and Supplementary Fig. 6a. This guarantees the absence of SPPs in the filtering mechanism because the electric fields are aligned parallel to the metallic interface. In fact, for the given structural design, the filtering function is lost or largely degraded when the white light source is p-polarized (Supplementary Fig. 6b). Furthermore, one can further see that the excited mode profiles do not represent that of surface plasmons, as the field is peaked in the middle of the nanowire rather than at the metal-TiO_x interface, as seen in Figure 1d. Moreover, since the filtering mechanism is not based on SPP interference, the periodicity does not control the color tunability.

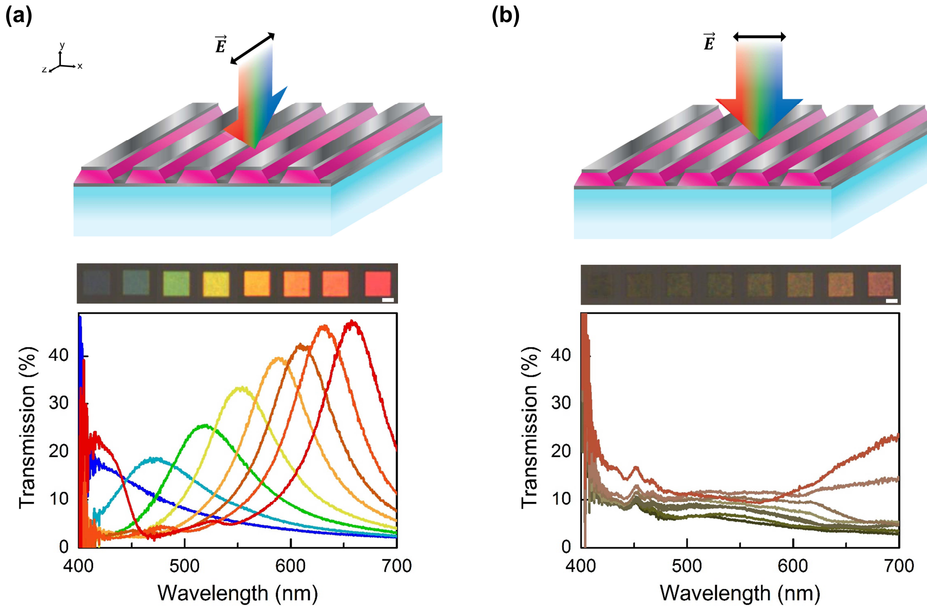

Supplementary Fig. 6. Polarization-dependent color pixels. From top to bottom: Schematic, OM images and transmission spectra of palette of pixels, with nanoresonators of fixed period

(400 nm) and different widths (70 to 310 nm), excited by (**a**) s- and (**b**) p-polarized white light. Scale bar is 5 μm.

Supplementary Note 4. Tunable gamut through the resist closure effect

In order to access the limits of sRGB colors, the spectral transmission peak position within the entire visible range (400~700 nm) must be sensitive to changes in the fabricated nanoresonator size. In our studies the fundamental resonance of the nanoresonator was used as it permits filtering at the smallest dimensions. To fully access the visible range, the fundamental mode profile must vary in size but maintain its spatial symmetry, suggesting that the TiO_x core should vary in both lateral and vertical dimensions. We employed a 'resist closure effect' in the TiO_x evaporation process to achieve this goal (Supplementary Fig. 7a).

Supplementary Fig. 7b describes the nanowire width-height relation for different amounts of evaporated TiO_x measured from cross-sectional images of the pixels. The total amount of evaporated TiO_x was quantified in terms of its thickness without the resist closure effect, equivalently expressed as the TiO_x film thickness. We fabricated three pixel sets with TiO_x film thicknesses of 68, 92, and 115 nm, denoted as L (low-), M (middle-) and H (high thickness) pixel sets, respectively, to provide width-height relations for variable amounts of deposited TiO_x. Polynomials were fitted onto each set of data points to model the evolution of nanowire shape from triangle to trapezoid. One can observe that at wide groove widths, the TiO_x nanowire height asymptotically approaches that of the TiO_x film (described by the dotted lines) as the groove width is too large for the resist closure effect to be significant. However, for narrow widths similar to or less than the deposited TiO_x film thickness, the groove can close prematurely, resulting in a triangular TiO_x nanowire of height shorter than that of the film. These two effects increase the degree of nonlinearity in the dependence of nanowire height on width, especially for larger amounts of deposited TiO_x, as can be observed from Supplementary Fig. 7b.

Different width-height trends give rise to different width-resonance characteristics including spectral range. Supplementary Fig. 7c illustrates the calculated transmission through a nanoresonator as a function of wavelength and width for the three pixel sets, based on models derived from polynomial fits of Supplementary Fig. 7b. Each model included a bare Ag background of fixed area in order to maintain consistent transmission efficiencies among nanoresonators of different widths. For comparison, a reference pixel set with no resist closure effect is also considered. In this case, the nanowire height was set to be invariable at 92 nm, corresponding to the film thickness of the M-pixel set. For the reference model and L-pixel set, exhibiting little or no variation in nanowire height, the spectral range of resonances is found to be limited to ~200 nm. This narrow range results from the inability of the enlarged resonant mode profiles formed with longer wavelengths to be accommodated within the vertical dimension of the nanowire. However, with variation in nanowire height as shown by the M and H-pixel sets, the spectral range can be expanded to more than 300 nm.

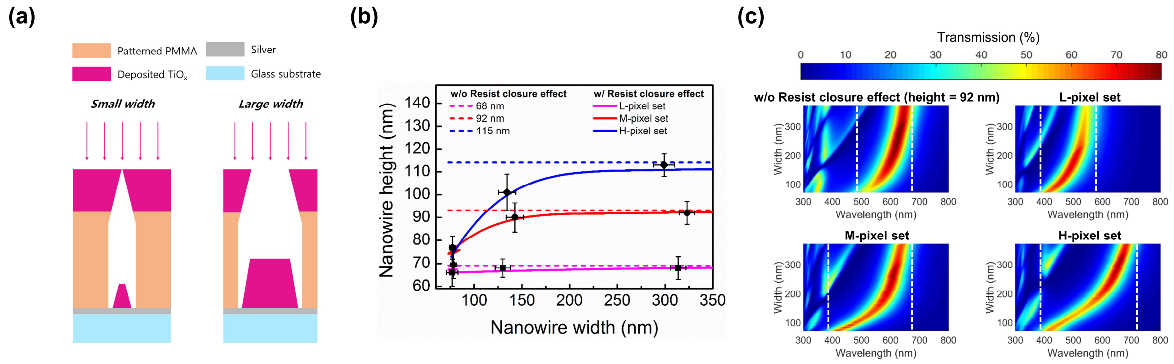

Supplementary Fig. 7. Tunable gamut through the resist closure effect. (**a**) Schematic of the resist closure effect. Creation of overhangs on the resist by the evaporated material results in the formation of triangular or trapezoidal structures in the groove for widths smaller or larger than the deposited material thickness, respectively. (**b**) Width-height relation of the deposited

TiO_x nanowire for different amounts of deposited material: L-pixel set = 68 nm, M-pixel set = 92 nm, and H-pixel set = 115 nm. Black dots refer to the measured nanowire dimensions from SEM images of FIB cross-sections. Solid lines refer to polynomial fits to the experimental data. Dashed lines represent rectangular nanowires with no resist closure effect. (**c**) Calculated transmission as a function of wavelength and width for the L, M, H-pixel sets and rectangular nanowires of height 92 nm with no resist closure effect.

Supplementary Note 5. Control of spectral range from single-color pixels

Control over the range of accessible resonances, or equivalently the variability in spectral range, through the deposited TiO_x film thickness was experimentally demonstrated with the L- and M-pixel sets in Supplementary Fig. 8 and H-pixel set in Figure 1e-g.

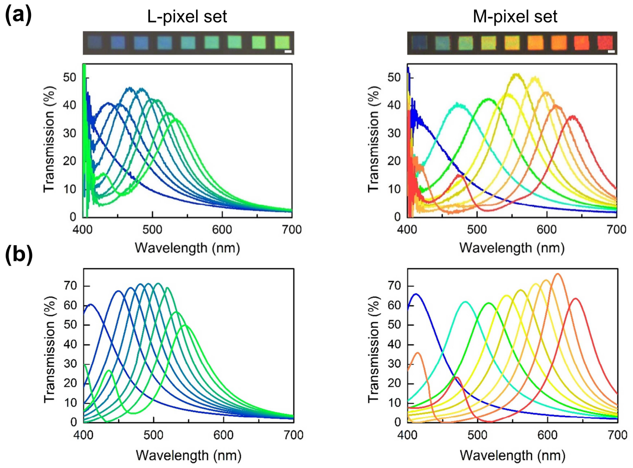

Supplementary Fig. 8. Control of spectral range from single-color pixels. (**a**) OM images and measured transmission spectra of the L and M-pixel set. Scale bar is 5 μm (**b**) Simulated transmission spectra of the L and M-pixel set.

Supplementary Methods. Quantitative assessment of wide gamut color pixels

Definition of the sRGB gamut coverage:

The sRGB gamut coverage is defined as the ratio between the area of a polygon with vertices defined by the pixel chromaticities and the full sRGB triangle area. The pixel chromaticities are derived from their absolute transmission spectra. An identical approach can also be found in Ref. 1 (*Light Sci. Appl*. **2017**, *6*, e17043).[1] If the polygon extends outside the sRGB triangle, it is truncated and only the area within the sRGB triangle is considered.

Assessment of gamuts by other groups and ours:

To build the scatter plot, we extracted the chromaticities from color diagrams of reported by other groups, and calculated their areal coverage. Some of these color diagrams were reported from multiple samples sharing the same design principle and strategy as described in Ref. 2 (*Sci. Rep.* **2017**, **7**, 40649),[2] rather than from a single sample. Therefore, to be consistent, and to assess the largest possible gamut coverage offered by each design principle, we applied the above approach of Ref. 2 to all reported gamuts as shown in Figure 3c.

For a fair comparison of our gamut coverage with those of other groups, we applied the same assessment approach as described above- involving three samples (L, M, and H pixel sets). The uniform and vibrant spectral responses over the entire visible range result in a wide-area polygon sharing large portions of the sRGB triangle borders and recording one of the largest sRGB gamut coverages (74.1% (± 0.9%)).

For practical purposes, we also added to the scatter plot the gamut coverage of a single sample—that of the M-pixel set. The M-pixel set alone is able to achieve a sRGB gamut coverage of ~69%, which is still considered as one of the largest in transmission mode.

Technical notes:

1. To achieve the maximum possible gamut area, chromaticities need to reside furthest away from the color diagram center. However, in some cases, certain chromaticities may reside closer to the center than its adjacent neighboring chromaticities. This is especially true for the purple color, which cannot be produced through a single transmission peak. For systems that cannot produce this color, including its chromaticity can decrease the gamut area. We can dispel such a possibility with our system because any missing point between two adjacent chromaticities can be created through a linear combination of the two hybrid nanoresonators. We introduce a purple pixel (denoted as the blue rhombus) in the M-pixel set as shown in Supplementary Fig. 9b, to illustrate this concept. With or without the purple pixel, the gamut coverage stays consistent.

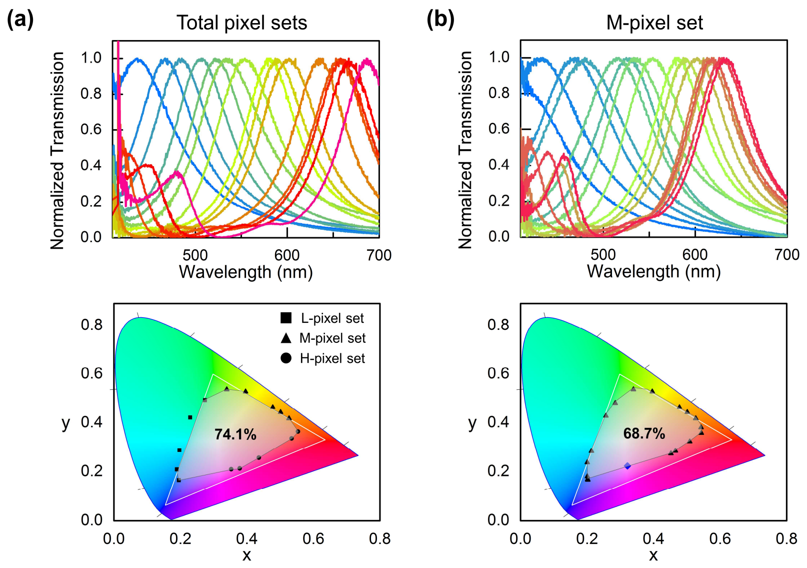

Supplementary Fig. 9. Normalized transmission spectrum and corresponding CIE 1931 chromaticity diagram for total pixel sets (a) and M-pixel set alone (b). The blue rhombus in **b** represents the chromaticity for a dual color pixel of two nanoresonators (width = 70 and

340 nm), and white line represents the sRGB triangle.

2. The expansion of the color gamut can be confirmed in the CIE chromaticity diagram of three pixel sets (L-, M-, and H- pixel sets), as shown in Supplementary Fig. 9a. For the L-pixel set, the filtered colors correspond to the left segment of the standard RGB (sRGB) triangle. This range is further extended to the right segment of the triangle with the M-pixel set. Full accessibility of the sRGB triangle including its bottom segment is achieved through the H-pixel set. In all cases, the chromaticities of the filtered light are positioned on the sRGB triangle borders, indicating that the pixels meet the basic requirements for color saturation and gamut in standardized display and imaging applications.

3. The shape of the color chromaticity polygon is an important figure-of-merit for assessing the full accessible range of visible colors. The quantitative values of gamut coverage alone may be insufficient for evaluating the range of colors. For example, some of the gap-plasmon (GPs) based pixels may produce inconsistent spectral forms across the entire visible wavelength range. Such pixels, for instance, may produce highly saturated blue colors, but desaturated red colors, resulting in an elongated chromaticity polygon in the CIE diagram. In this case, even though the gamut coverage value itself can be large due to the saturated blue colors, such designs are still incapable of rendering vivid red colors. This can function as a significant deteriorating factor in achieving high-resolution hyperspectral/multispectral imaging at all visible wavelengths.

We emphasize that, in our study, vibrant red, green and blue primaries can be accessed, preventing the color polygon from being severely skewed or distorted. Both the size (~74% of sRGB gamut coverage) and the shape of the achieved color space satisfy the ideal criterion for accomplishing hyperspectral/multispectral imaging over all visible wavelengths.

Supplementary Note 6. Demonstration of linearity

As shown in Supplementary Fig. 10a, we designed a series of pixels labeled from 1 to 7, composed of different ratios of two distinct nanoresonators with widths (colors) of 100 (blue) and 340 nm (red). The dotted box represents the smallest repeating unit. SEM images portraying representative regions of the fabricated pixel are shown in the bottom panel.

We can define the basis functions of our system as the measured transmission spectra of the full single color pixels, represented by the solid curves in Supplementary Fig. 10b and c for the blue and red colors, respectively. The dotted curves represent different weighting coefficients multiplied to the basis function. The superposition principle can be demonstrated by comparing the measured transmission spectrum from a dual color pixel from either one of the 2 to 6 pixel combinations shown in Supplementary Fig. 10a with the linearly combined basis functions multiplied by the corresponding weighting coefficients. For example, the mixing ratio for the blue and red nanoresonators for pixel 2 is 3:1, corresponding to a weighting coefficient of 0.75 to 0.25, respectively. For pixel 2 to be a linear system, the measured transmission spectrum must be equivalent to the addition of basis functions of the blue and red pixels multiplied by 0.75 and 0.25, respectively. Supplementary Fig. 10d shows that indeed the two curves are equivalent within an experimental range of error, establishing the linearity of the nanoresonators.

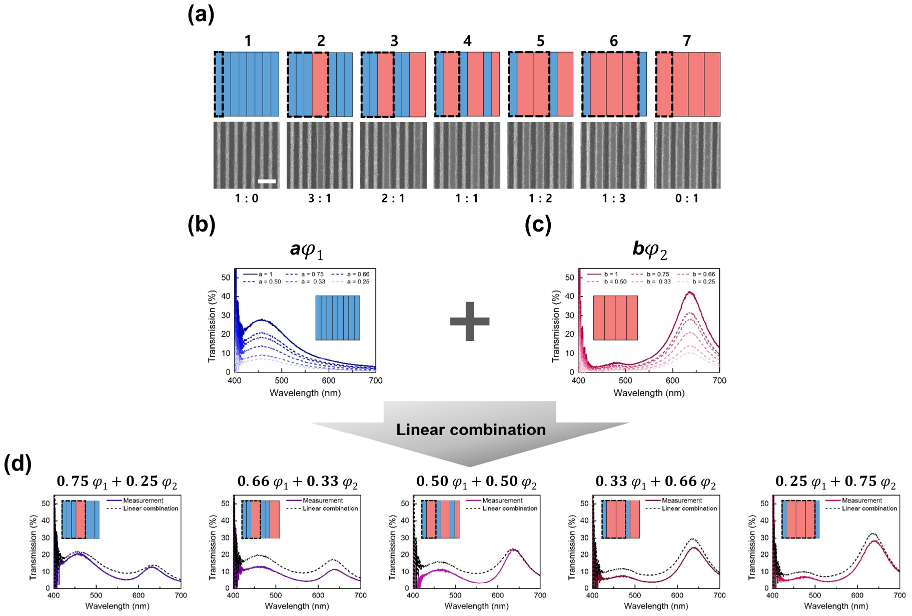

Supplementary Fig. 10. Demonstration of linear combination for dual-color pixels. (**a**) Schematic (top) and SEM images (bottom) of a series of dual-color pixels labeled from 1 to 7, composed of different ratios of two distinct nanoresonators with widths of 100 nm and 340 nm. The dashed box represents the smallest repeating unit. Scale bar is 500 nm. Transmission spectra with different weighing coefficients for (**b**) blue and (**c**) red pixels. The solid line represents the measured spectrum of a single-color pixel (i.e., the basis function), and the dotted lines represent the basis function multiplied by sub-unity coefficients, respectively. (**d**) Measured transmission spectra of the 2, 3, 4, 5, and 6 dual-color pixels (solid line) and linearly combined spectra from the two basis functions each multiplied by the corresponding weighing coefficients (dotted lines).

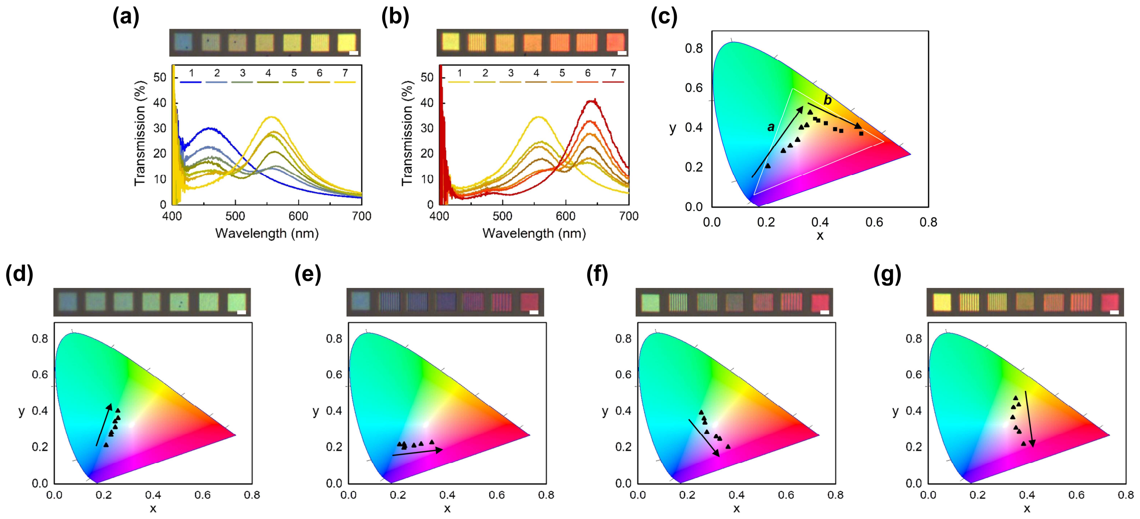

Supplementary Fig. 11. Additional dual-color pixels from linear combinations of two nanoresonators. (a, b) OM images and measured transmission spectra of dual-color pixels configured according to the series shown in **Figure 2a** with the 1 and 7 pixel corresponding to (**a**) blue and yellow (nanoresonator width = 100 and 160 nm, respectively) and (**b**) yellow and red colors (nanoresonator width = 250 nm). (**c**) CIE chromaticity diagram representing the chromaticities derived from the measured spectra of **a-b**. (**d-g**) OM images and CIE chromaticities of additional dual-color pixels consisting of repeating units of two distinct nanoresonators with different ratios. The widths of the distinct nanoresonators are (**d**)100 and 130 nm, (**e**) 100 and 400 nm, (**f**) 130 and 340 nm, and (**g**) 160 and 340 nm. Scale bar is 5 μm.

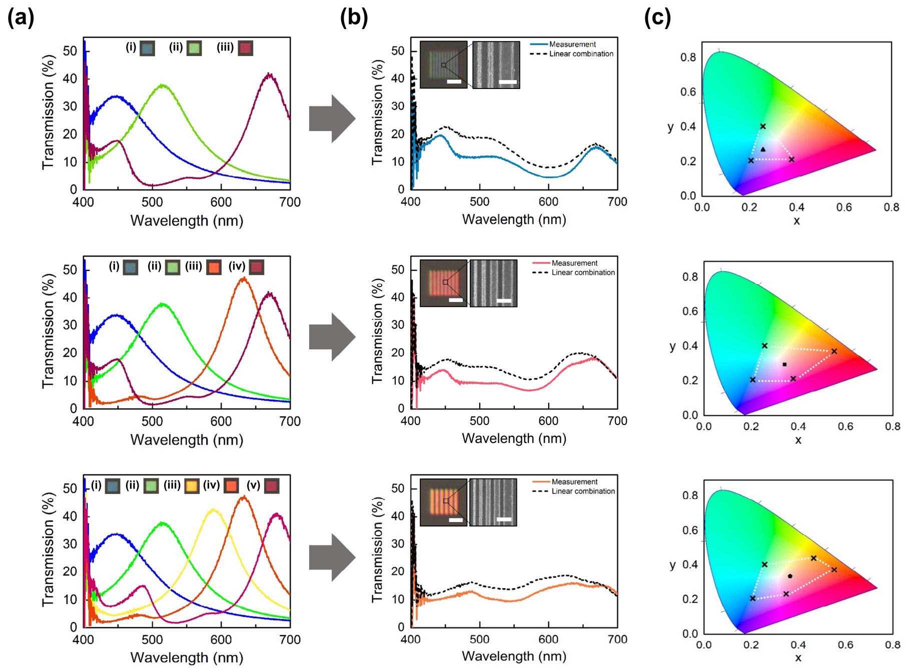

Supplementary Fig. 12. Demonstration of multicolor pixels from three (top row), four (middle row) and five (bottom row) distinct nanoresonators. (**a**) Transmission spectra of the distinct nanoresonators measured from single-color pixels. Inset: OM images of the pixels. (**b**) Measured transmission spectrum of multicolor pixel (solid lines) composed of the distinct nanoresonators at unity ratio, and average of the spectra shown in **a** (dotted lines). Inset: OM and SEM images of corresponding multicolor pixel. Scale bar is 5 μm and 100 nm, respectively (**c**) CIE chromaticity diagram representing the mixed color of multicolor pixel positioned at the center of color polygons defined by individual 'pure' colors from distinct nanoresonators. The nanoresonators widths for each multicolor pixel are (top row) 100 nm, 130 nm and 340 nm (middle row) 100 nm, 130 nm, 250 nm, and 340 nm, (bottom row) 100 nm, 130 nm, 190 nm, 250 nm, and 400 nm.

Supplementary Note 7. Decoupling gamut from spatial resolution

It is important to depict the relationship between DPI and gamut coverage, since the main objective of this study is to simultaneously achieve high spatial resolution (i.e., DPI) and spectral resolution (gamut coverage), which has been well recognized as a crucial challenge and unmet need in the structural color community. Even as the size of pixels (i.e., number of nanoresonators) decreases, the spectral peak shape and thereby the color gamut are maintained. In Supplementary Fig. 13, we present the normalized simulated transmission spectra of blue, green and red pixels as the number of nanoresonators increase from 1 to infinity (array). No significant change in spectral behavior is observed for the dipolar response. We also confirm this behavior experimentally, as shown in Supplementary Fig. 14, where nanoresonators of widths from 80 to 340 nm with a fixed interspacing of 100 nm show consistent spectral responses for various pixel sizes. One can see that our hybrid pixel design preserves the spectral purity of the filtered light even when the number of elements reduces to unity, demonstrating the ability to decouple the gamut from spatial resolution.

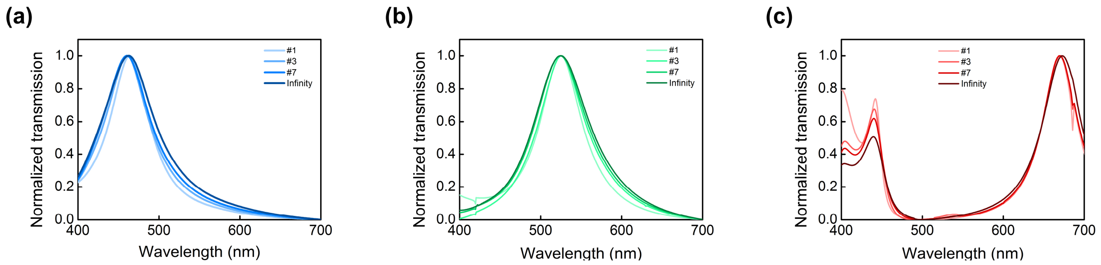

Supplementary Fig. 13. Normalized calculated transmission spectra from (**a**) blue, (**b**) green and (**c**) red pixels with different number of nanoresonators**.**

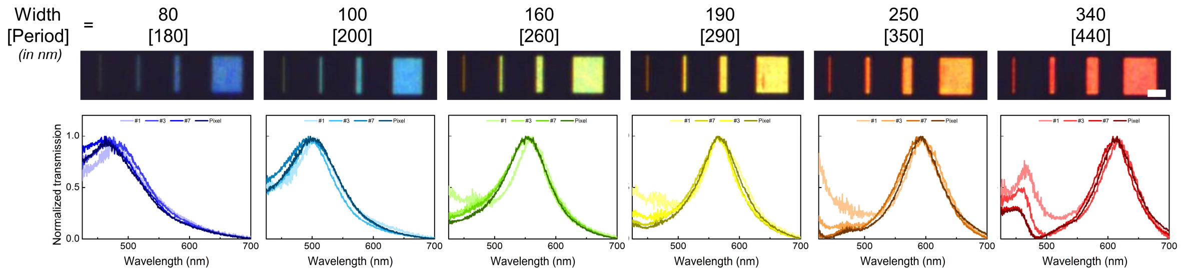

Supplementary Fig. 14. Optical microscope (top row) and normalized transmission spectrum (bottom row) of full visible color pixels as the nanoresonator widths vary from 80 (most left column) to 340 nm (most right column) at a fixed interspacing of 100 nm. In each color pixel sets, the number of nanoresonators vary from 1 (single elements) to ≥20 (10 µm-sized pixels). Scale bar is 5 µm.

	Color Gamut[a)] (%)	Absolute Transmission of Arrays (%)	Periodicity Independence	Smallest Pixel Size [Smallest Repeating Unit][b)] (nm)	Maximum Single-color Dots-per-inch (dpi)[c)]	Size-invariant Linear Color Mixing Capability
**This work [Total pixel sets] (Ewha Womans Univ.)**	**74.1 ± 0.8[d)]**	**42.1 ± 6.8[d)]**	**Yes**	**80 [180]**	**141,000**	**Yes**
**This work [M-pixel set] (Ewha Womans Univ.)**	**68.7 ± 1.1[d)]**	**43.9 ± 7.8**	**Yes**	**80 [180]**	**141,000**	**Yes**
Nat. Commun., 2010 (Univ. of Michigan)[3]	N.A	52.5±7.5	No	350 [540]	47,000	No
Adv. Funct. Mat., 2017 (Univ. of Glasgow)[4]	73.4 ± 0.7	Difficult to define[e)]	No	370 [500]	50,800[f)]	No
Sci. Rep., 2013 (Lehigh Univ.)[5]	N.A	Difficult to define[e)]	No	405 [540]	47,000	No
ACS Nano, 2015 (Univ. of Glasgow)[6]	42.5 ± 0.4[g)]	7.2±6.1	No	370 [500][h)]	50,800[f)]	No
ACS Nano, 2017 (EPFL)[7]	13.6± 0.6	Difficult to define[e)]	No	900 [1,080]	23,500	No
Sci. Rep., 2017 (Korea Univ.)[2]	47.7 ± 0.7[i)]	34.5±5.5	No	350 [460][h)]	55,200	No
Adv. Opt. Mat., 2016 (Fuzhou Univ.)[8]	9.7 ± 0.3	Difficult to define[e)]	No	200 [270]	94,100	No
Appl. Phys. Lett., 2011 (Toyota.Inc)[9]	N.A	25.1 ± 12.2[j)]	No	N.A	N.A	No
Optics Express, 2010 (Univ. of Glasgow)[10]	N.A	35.2±7.2	No	840 [990]	25,700	No
Sci. Rep., 2016 (La Trobe University)[11]	51.7± 1.2[k)]	6.6±3.4	No	440 [560]	45,400	No
ACS Nano, 2013 (Caltech)[12]	N.A	47.5±2.5	No	N.A	N.A	No
Sci. Rep., 2014 (Univ. of Melbourne)[13]	N.A	7.5±2.5	Yes	392 [430]	59,100	No
ACS Nano, 2015 (ETH)[14]	25.6± 0.9[l)]	42.5 ± 37.5	No	N.A	N.A	No
Nano Lett., 2012 (Caltech)[15]	N.A	45.2±5.1	Yes	1,200 [1,500]	17,000	No

Supplementary Table 1. Comparison of color filtering performance in transmission mode.[2-15]

a) Calculated in CIE 1931 space as "maximum area defined by measured chromaticities / area of sRGB triangle".

b) Smallest repeating unit refers to the total period (structure dimension + interspacing gap).

c) Calculated as one-dot-per-smallest repeating unit.

d) CIE chromaticities and transmission intensities of L-, M-, and H-pixel sets are considered.

e) Transmission spectra are based on CMYK color system, which makes it difficult to define absolute transmission efficiency.

f) DPI under static mode (i.e., single polarization mode) is considered.

g) CIE chromaticities of three pixel sets with Al thickness of 75, 100 and 125 nm in both TM and TE modes are considered.

h) Two periods are considered as the smallest repeating unit for metallic hole structures.

i) CIE chromaticities of both D- and H- PCF are considered.

j) Transmission intensity of both triangular and circular holes are considered.

k) CIE chromaticities at all polarization modes (TM, 45º, and TE) are considered.

l) CIE chromaticities of three samples with evaporation angles of 20º, 30º, and 40º are considered.

Supplementary Note 8. Transmission from incident light of different angular distributions

We verified the invariance of the transmitted response of a representative hybrid nanoresonator for incident light of different angular distributions. Supplementary Fig. 15 shows the simulated normalized transmission spectrum of a green pixel under plane wave illumination and Gaussian beam illumination modelling light through a lens with different NA values (0.2, 0.5 and 0.9). Despite the large angular variation, the spectral position and form of the transmission peak stays relatively consistent.

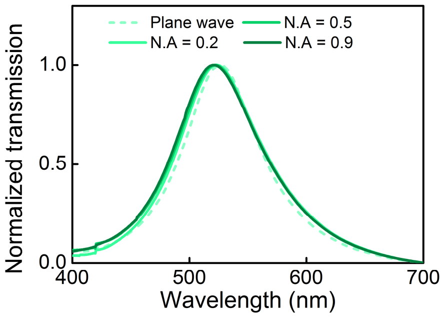

Supplementary Fig. 15. Calculated transmission spectra of hybrid nanoresonators under plane wave illumination and Gaussian beam illumination modelling light through a lens with 0.2, 0.5 and 0.9 NA.

Supplementary Note 9. Transmission response of color pixels with hybrid nanoresonators of different lengths

We simulated the transmission of hybrid nanoresonators as a function of nanoresonator lengths from 0.5 μm to infinity, when the nanoresonator width is 100 nm and interspacings between neighboring nanoresonators in both axis are 150 nm under plane wave and Gaussian beam illumination (Supplementary Fig. 16). As the aspect ratio of the nanowire decreases, the response deteriorates, as contributions from the edges increase. Gap-plasmonic responses can also appear leading to loss of spectral purity or color saturation.

Although degraded compared to the case of a long aspect-ratio nanoresonator, the transmitted response at a nanoresonator length of 0.5 μm (aspect ratio 5:1) still retains the overall spectral form, with the transmission maximized at the resonant wavelength (~ 490 nm). Such unchanging spectral characteristics at different nanoresonator lengths are also observed under a high numerical aperture. This shows that the packing density in the transversal direction can be increased, but at the expense of reduced intensity and sRGB gamut coverage.

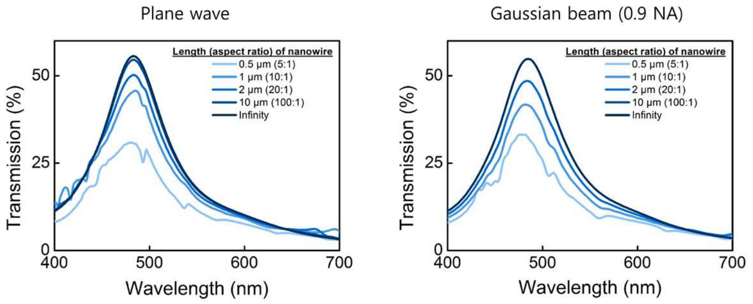

Supplementary Fig. 16. Transmission spectra of hybrid color pixels with different lengths of hybrid nanoresonators under (left) plane wave and (right) Gaussian beam (0.9 NA) illumination.

Supplementary References

1 Chen, H. W. *et al.* Going beyond the limit of an LCD's color gamut. *Light Sci. Appl.* **6**, e17043, (2017).

2 Lee, S. U. & Ju, B. K. Wide-gamut plasmonic color filters using a complementary design method. *Sci. Rep.* **7**, 40649, (2017).

3 Xu, T., Wu, Y. K., Luo, X. & Guo, L. J. Plasmonic nanoresonators for high-resolution colour filtering and spectral imaging. *Nat. Commun.* **1**, 59, (2010).

4 Heydari, E., Sperling, J. R., Neale, S. L. & Clark, A. W. Plasmonic color filters as dual-state nanopixels for high-density microimage encoding. *Adv. Funct. Mater.* **27**, 1701866, (2017).

5 Zeng, B., Gao, Y. & Bartoli, F. J. Ultrathin nanostructured metals for highly transmissive plasmonic subtractive color filters. *Sci. Rep.* **3**, 2840, (2013).

6 Li, Z. B., Clark, A. W. & Cooper, J. M. Dual color plasmonic pixels create a polarization controlled nano color palette. *ACS Nano* **10**, 492-498, (2016).

7 Wang, H. *et al.* Full color generation using silver tandem nanodisks. *ACS Nano* **11**, 4419-4427, (2017).

8 Yang, C. *et al.* Angle robust reflection/transmission plasmonic filters using ultrathin metal patch array. *Adv. Opt. Mater.* **4**, 1981-1986, (2016).

9 Inoue, D. *et al.* Polarization independent visible color filter comprising an aluminum film with surface-plasmon enhanced transmission through a subwavelength array of holes. *Appl. Phys. Lett.* **98**, 093113, (2011).

10 Chen, Q. & Cumming, D. R. S. High transmission and low color cross-talk plasmonic color filters using triangular-lattice hole arrays in aluminum films. *Opt. Express* **18**,

14056-14062, (2010).

11 Balaur, E., Sadatnajafi, C., Kou, S. S., Lin, J. & Abbey, B. Continuously tunable, polarization controlled, colour palette produced from nanoscale plasmonic pixels. *Sci. Rep.* **6**, 28062, (2016).

12 Burgos, S. P., Yokogawa, S. & Atwater, H. A. Color imaging via nearest neighbor hole coupling in plasmonic color filters integrated onto a complementary metal-oxide semiconductor image sensor. *ACS Nano* **7**, 10038-10047, (2013).

13 Rajasekharan, R. *et al.* Filling schemes at submicron scale: Development of submicron sized plasmonic colour filters. *Sci. Rep.* **4**, 6435, (2014).

14 Duempelmann, L., Casari, D., Luu-Dinh, A., Gallinet, B. & Novotny, L. Color rendering plasmonic aluminum substrates with angular symmetry breaking. *ACS Nano* **9**, 12383-12391, (2015).

15 Yokogawa, S., Burgos, S. P. & Atwater, H. A. Plasmonic color filters for CMOS image sensor applications. *Nano Lett.* **12**, 4349-4354, (2012).
